# Child Poverty Trends by Race and Ethnicity in the US From 2022 to 2025

**DOI:** 10.1001/jamapediatrics.2025.5630

**Published:** 2026-01-12

**Authors:** Haris Majeed, Carmen H. Logie, Daniyal Zuberi

**Affiliations:** 1 University of Toronto, Toronto, Ontario, Canada

## Abstract

This cross-sectional study examines race- and ethnicity-based trends in child poverty in the US from 2022 to 2025.

The prevalence of child poverty (defined as age <18 years living in families with low income) has been a concern in the US for several decades. The US places second only to Romania in rankings of child poverty rates among 35 high-income countries.^1^

Prior to the COVID-19 era, child poverty was reduced in the US, yet after the expiration of the 2021 US federal government expansion of the Child Tax Credit during the COVID-19 pandemic as part of the American Rescue Plan income provisions, the child poverty rate doubled and returned to exceed that of most other high-income countries, particularly for racial and ethnic minority children.^2^ While substantial research has examined child poverty trends in the US, there remains an urgent need to understand race- and ethnicity-based trends after the COVID era (ie, from 2022 onwards).

## Methods

This cross-sectional study used annual race- and ethnicity-specific child poverty prevalence for each available county, ascertained from the County Health Rankings (funded by the Robert Wood Johnson Foundation)^3^ and spanning the post–COVID-19 era, for years 2022 and 2025 (eTable in Supplement 1). The study was based on secondary analysis of publicly available unrestricted data (deidentified). As such, the research does not involve human participants, and institutional review board approval and informed consent were not required. The Strengthening the Reporting of Observational Studies in Epidemiology (STROBE) reporting guidelines were followed. The County Health Rankings uses US Census Bureau data, with support from other federal agency programs to provide more current estimates. At the county level, the data included estimates on children aged 5 to 17 years with families in poverty, children younger than 18 years in poverty, all people in poverty, and median household income. We included 2 racial categorizations (non-Hispanic Black and non-Hispanic White [hereafter referred to as Black and White]) and 1 ethnicity (Hispanic). Other racial and ethnic groups were not included due to small sample sizes. Child poverty prevalence rates were included for each of the 48 contiguous US states (all [N = 3066 counties]; Black [n = 1694 counties]; Hispanic [n = 2469 counties]; and White [n = 2668 counties]) (eFigure in Supplement 1). Child poverty prevalence was defined as the percentage of individuals younger than 18 years in poverty. The data were collected on random-digit telephone surveys conducted annually in all states, counties, and US territories; all measures are based on self-report.^3^

To determine county-level and race-specific trends, the annual percentage change (APC) was computed between the years of 2022 and 2025. Data were shown by census region (Midwest, Northeast, South, and West), as well as on a geographical heat map.

The *t* test was used to test statistical significance between racial and ethnic groups, and 2-tailed *P* < .05 was deemed significant. All statistics and computations were performed using RStudio version 4.3.1 (R Foundation).

## Results

Overall, from 2022 to 2025, child poverty in the US ranged from an absolute minimum of 2.6% (West) to a maximum of 63.2% (South). Nearly all included races and ethnicities throughout US regions experienced a reduction in child poverty in post-COVID years (Table), a trend also observed geographically (Figure). The highest concentration of poverty was noted in Southeastern counties. Hispanic and White populations experienced significant declines, whereas no significant trends were found for Black respondents except in the South (Table). The strongest significant reduction was observed in Hispanic individuals residing in the Northeast US (APC, −14.1%; 95% CI, −20.1% to −6.5%; *P* < .001) (Table). Compared to White children, child poverty rates for Black and Hispanic children were significantly greater in both 2022 and 2025 across all regions, where some counties in the US had excessive rates greater than 98%.

**Table.** Trends in Child Poverty Rates From 2022 to 2025, Stratified by US Region and Race and Ethnicity

Region	Race and ethnicity	Child poverty rate, % (95% CI)		APC (95% CI), %	*P* value^a^
		2022	2025		
Midwest	Hispanic	26.1 (24.9 to 27.3)	24.1 (22.9 to 25.2)	−7.8 (−12.7 to −3.1)	.001
	Non-Hispanic Black	36.6 (34.3 to 38.8)	35.2 (32.9 to 37.4)	−3.9 (−9.2 to 2.7)	.28
	Non-Hispanic White	12.9 (12.5 to 13.4)	12.2 (11.7 to 12.6)	−6.0 (−7.9 to −3.5)	<.001
Northeast	Hispanic	26.2 (24.2 to 28.2)	22.5 (20.7 to 24.3)	−14.1 (−20.1 to −6.5)	<.001
	Non-Hispanic Black	30.4 (27.7 to 33.1)	28.4 (25.8 to 31.1)	−6.4 (−17.7 to 0.3)	.06
	Non-Hispanic White	12.3 (11.5 to 13.1)	11.5 (10.8 to 12.3)	−6.4 (−8.2 to −2.1)	.001
South	Hispanic	32.9 (31.8 to 34.0)	28.8 (27.8 to 29.9)	−12.3 (−13.7 to −7.4)	<.001
	Non-Hispanic Black	37.7 (36.6 to 38.8)	36.9 (35.7 to 38.0)	−2.2 (−5.6 to −0.4)	.02
	Non-Hispanic White	16.2 (15.7 to 16.7)	15.5 (15.0 to 16.0)	−4.4 (−7.4 to −2.7)	<.001
West	Hispanic	24.3 (23.0 to 25.7)	22.5 (22.1 to 23.9)	−7.6 (−17.2 to −5.5)	<.001
	Non-Hispanic Black	25.9 (22.9 to 28.9)	26.3 (23.4 to 29.3)	1.8 (−15.3 to 10.7)	.73
	Non-Hispanic White	12.8 (11.9 to 13.7)	11.8 (11.0 to 12.6)	−8.0 (−10.7 to 1.5)	.14

Abbreviation: APC, annual percentage change.

^a^ Significance was based on the mean differences in child poverty rates for Hispanic and non-Hispanic Black children, which were consistently and substantially higher than for non-Hispanic White children in both 2022 and 2025. Computed using *t* tests.

**Figure.**
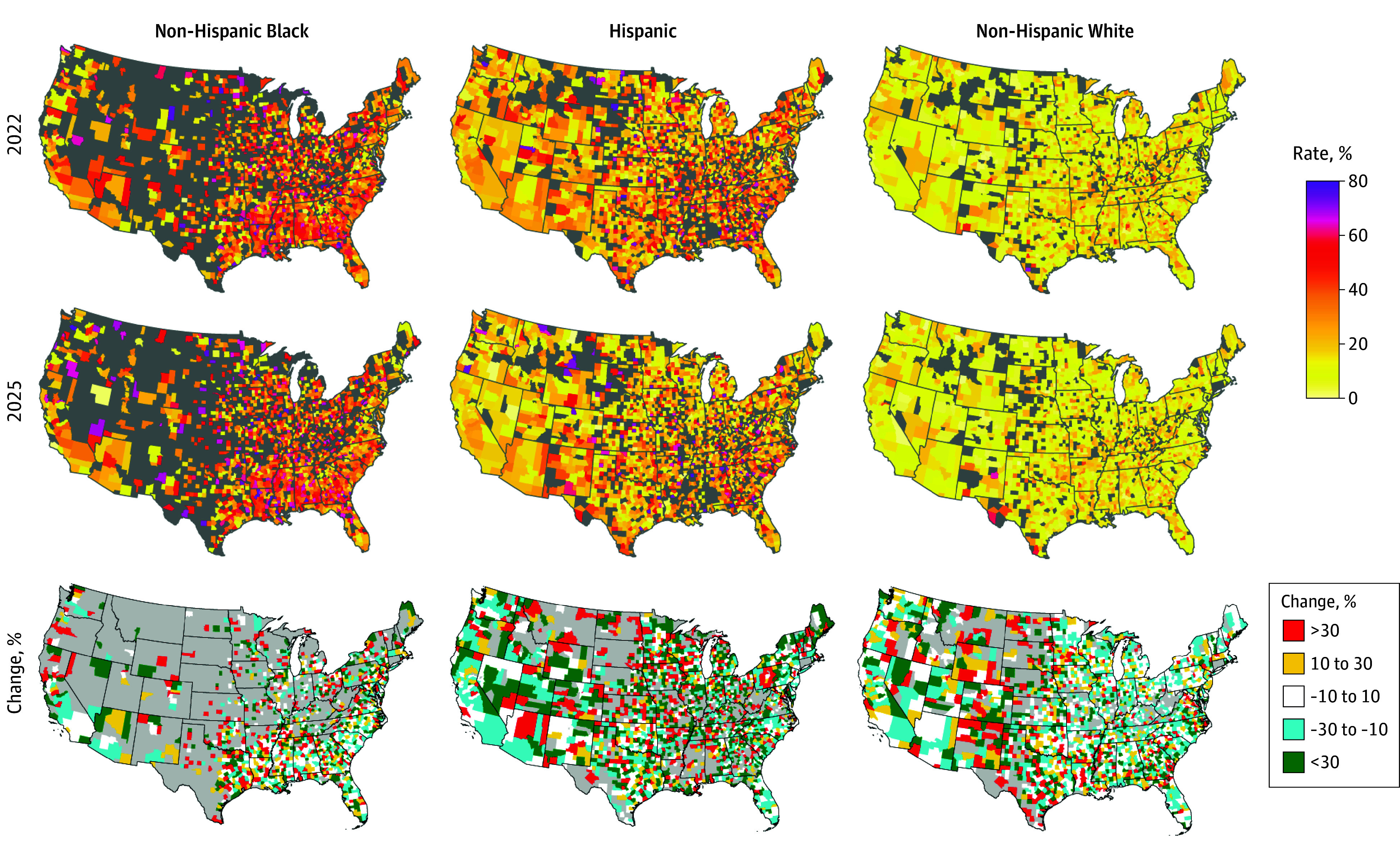
County-Specific Geographic Illustration of Race- and Ethnicity-Specific Child Poverty Rates for 2022 and 2025 and Percentage Change Between the 2 Years Gray areas denote missing county data.

## Discussion

To our knowledge, this is one of the first studies to document recent racial- and ethnic-specific child poverty trends after the COVID era. Overall, from 2022 to 2025, most counties experienced a population-level decline in child poverty rates, with rates for Black and Hispanic children experiencing the greatest changes. Despite overall decline, Black and Hispanic children continued to experience disproportionately higher poverty rates compared with White children. We also found that regardless of race and ethnicity, child poverty trends in the Western counties have risen.

Child poverty is important to reduce, and one of the first sustainable development goals is to end poverty by 2030, which remains a major challenge based on our findings. Recent studies have revealed that poverty and resilience research has increased, particularly in the last 4 years; however, it will take significant additional effort from all levels of government to mitigate poverty. The association between economic growth and relative child poverty shows that economic growth is related to reductions in relative child poverty when combined with sufficiently extensive government transfers, while the opposite effect was found in the face of inadequate levels of government cash transfer systems.^4^ The stark differences in baseline poverty rates strongly suggest that these disparities are possibly rooted in structural racism rather than individual circumstances. These structural factors manifest regionally through persistent housing segregation, labor market discrimination leading to lower intergenerational wealth transfer, and inequitable distribution of resources for education and health services. Other reasons for the rise in child poverty in Western US regions could be due to changing demographic and immigration patterns in this region, with an increasing number of families lacking resources to provide for their children’s well-being,^5^ hence contributing to a rise in poverty. Likewise, the significant reductions observed for Hispanic children in the Northeast and South may reflect the profound yet fragile impact of recent federal policy reforms, such as the temporary expansion of the Child Tax Credit.

Furthermore, the US inflation rate shot to its peak around mid-2022, and from January 2020 to January 2024, regular food prices in Europe and the US increased by 15% to 23%.^6^ This may have exacerbated the material hardships driving child poverty.

Some limitations of this cross-sectional study include disproportion in the sample size of children surveyed for each county; less than 100% of children in each county were captured. Possible misclassification of interracial or interethnic identities may also affect sample sizes. Also, the timing of the actual surveys conducted may not correspond exactly with the years 2022 or 2025. Another limitation is the inability to conduct reliable analyses across all racial and ethnic populations (such as Asian and Pacific Islander) in the US, as the survey methodology resulted in limited sample sizes within a majority of counties. Ultimately, we propose that further regionally tailored policies are needed to reduce child poverty in the US.

## Notes

Supplement 1. eTable. Child Poverty Rates (%) for All Races Combined in the US, Spanning 2022-2025 eFigure. Box Plots for Child Poverty Rates (%) for Various Races, 2022 and 2025

Supplement 2. Data Sharing Statement
