# Supplementary material for: Child Poverty Trends by Race and Ethnicity in the US From 2022 to 2025
Source: JAMA Pediatr. 2026 Jan 12;180(3):336–9. doi: 10.1001/jamapediatrics.2025.5630 (PMC12797124; doi:10.1001/jamapediatrics.2025.5630)
Supplement: Supplement 1. — eTable. Child Poverty Rates (%) for All Races Combined in the US, Spanning 2022-2025 eFigure. Box Plots for Child Poverty Rates (%) for Various Races, 2022 and 2025 [file jamapediatr-e255630-s001.pdf]

Supplemental Online Content

Majeed H, Logie CH, Zuberi D. Child poverty trends by race and ethnicity in the US from 2022 to 2025. *JAMA Pediatr*. Published online January 12, 2026. doi:10.1001/jamapediatrics.2025.5630

eTable. Child Poverty Rates (%) for All Races Combined in the US, Spanning 2022-2025

eFigure. Box Plots for Child Poverty Rates (%) for Various Races, 2022 and 2025

This supplemental material has been provided by the authors to give readers additional information about their work.

© 2025 Majeed H et al. JAMA.Pediatrics.

eTable. Child Poverty Rates (%) for All Races Combined in the US, Spanning 2022-2025

Race	Region	Minimum	Mean (±SD)	Median	Maximum
Overall	Midwest	3.2	15.3±6.1	14.4	55.7
	Northeast	3.9	14.7±5.1	14.6	35.5
	South	2.8	22.9±8.3	22.3	63.2
	West	2.6	16.4±6.9	15.5	44.4
NH Black	Midwest	0.4	35.9±21.8	34.5	98.2
	Northeast	1.3	29.4±17.4	27.5	83.3
	South	0.6	37.3±18.1	36.9	98.4
	West	0.7	25.8±16.5	24.0	85.7
Hispanic	Midwest	0.8	25.1±16.8	22.1	88.5
	Northeast	0.5	24.3±13.8	22.6	74.4
	South	0.4	30.9±18.6	27.1	99.4
	West	0.5	23.1±13.0	20.8	80.6
NH White	Midwest	0.3	12.6±6.4	11.6	40.2
	Northeast	0.8	11.9±5.8	11.3	31.4
	South	0.2	15.8±8.9	14.5	81.8
	West	0.4	12.1±8.2	10.9	84.3

© 2025 Majeed H et al. JAMA.Pediatrics.

eFigure. Box Plots for Child Poverty Rates (%) for Various Races, 2022 and 2025

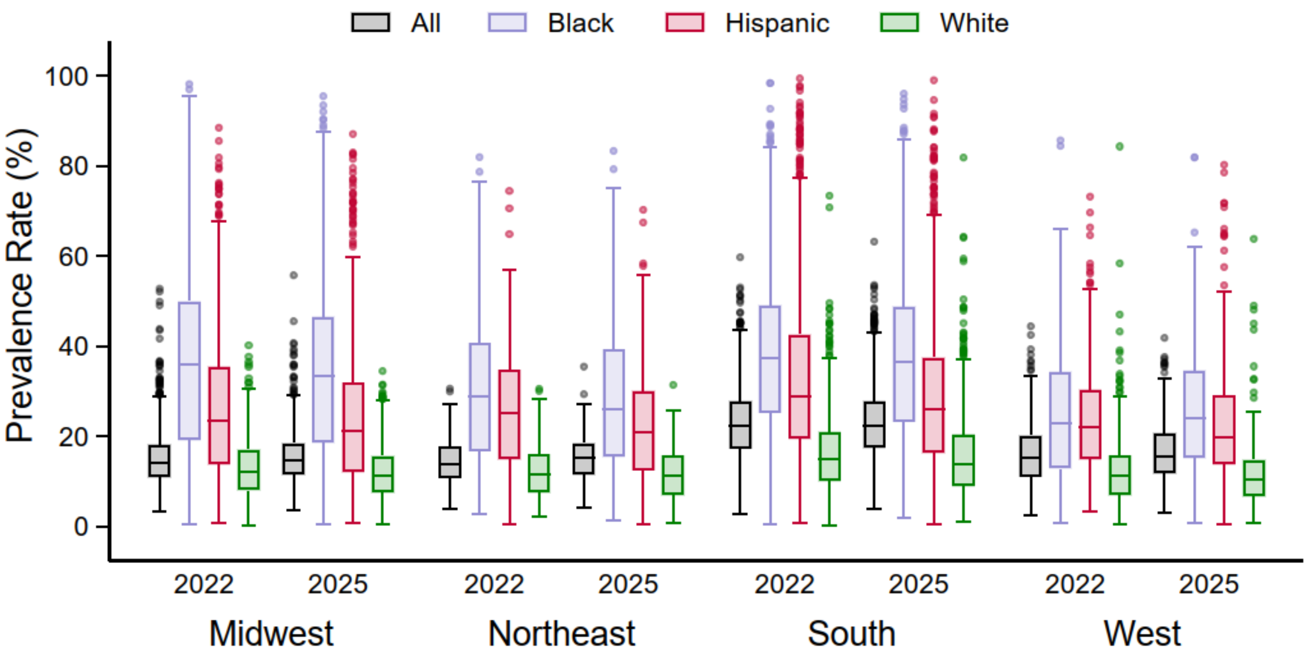

© 2025 Majeed H et al. JAMA.Pediatrics.
