# Supplementary material for: Child Poverty Trends by Race and Ethnicity in the US From 2022 to 2025
Source: JAMA Pediatr. 2026 Jan 12;180(3):336–9. doi: 10.1001/jamapediatrics.2025.5630 (PMC12797124; doi:10.1001/jamapediatrics.2025.5630)
Supplement: Supplement 2. — Data Sharing Statement [file jamapediatr-e255630-s002.pdf]

Data Sharing Statement

Majeed. Child Poverty Trends by Race and Ethnicity in the US From 2022 to 2025. *JAMA Pediatr*. Published January 12, 2026. doi:10.1001/jamapediatrics.2025.5630

Data
Data available: Yes
Data types: Deidentified participant data
How to access data: All data is available from the Robert Wood Johnson Foundation (https://www.countyhealthrankings.org/)
When available: With publication

Supporting Documents
Document types: None

Additional Information
Who can access the data: All included in research letter
Types of analyses: Any purpose
Mechanisms of data availability: Publicly available
Any additional restrictions: None
